# Development of a Miniaturized 2-Joule Pulsed Plasma Source Based on Plasma Focus Technology: Applications in Extreme Condition Materials and Nanosatellite Orientation

**DOI:** 10.3390/mi15091123

**Published:** 2024-09-01

**Authors:** Leopoldo Soto, Cristian Pavez, José Pedreros, Jalaj Jain, José Moreno, Patricio San Martín, Fermín Castillo, Daniel Zanelli, Luis Altamirano

**Affiliations:** 1 Center for Research and Applications on the Intersection of Plasma Physics, Matter and Complexity, P2mc, Comisión Chilena de Energía Nuclear, Santiago 7600713, Chile; cristian.pavez@cchen.cl (C.P.); jalaj.jain@cchen.cl (J.J.); jose.moreno@cchen.cl (J.M.); zanelli.93@gmail.com (D.Z.); 2 Departamento de Ciencias Físicas, Universidad Andres Bello, Republica 220, Santiago 8370134, Chile; 3 Departamento de Ingeniería Eléctrica, Universidad de Chile, Santiago 8370451, Chile; josepedreros@gmail.com; 4 Research Reactors and Nuclear Fuel Department, Comisión Chilena de Energía Nuclear, Santiago 7600713, Chile; patricio.sanmartin@cchen.cl; 5 Instituto de Ciencias Físicas, Universidad Nacional Autónoma de México, Cuernavaca 62210, Mexico; ciro@fis.unam.mx; 6 Dicontek Ltda., Santiago 7550171, Chile; laltamirano@dicontek.com

**Keywords:** dense plasma focus, portable pulsed plasma source, neutron pulses, X-ray pulses, particle beams, plasma shock, plasma facing materials, nuclear fusion, pulsed plasma thruster

## Abstract

Plasma focus devices represent a class of hot and dense plasma sources that serve a dual role in fundamental plasma research and practical applications. These devices allow the observation of various phenomena, including the z-pinch effect, nuclear fusion reactions, plasma filaments, bursts, shocks, jets, X-rays, neutron pulses, ions, and electron beams. In recent years, considerable efforts have been directed toward miniaturizing plasma focus devices, driven by the pursuit of both basic studies and technological advancements. In this paper, we present the design and construction of a compact, portable pulsed plasma source based on plasma focus technology, operating at the ~2–4 Joule energy range for versatile applications (PF-2J: 120 nF capacitance, 6–9 kV charging voltage, 40 nH inductance, 2.16–4.86 J stored energy, and 10–15 kA maximum current at short circuit). The components of the device, including capacitors, spark gaps, discharge chambers, and power supplies, are transportable within hand luggage. The electrical characteristics of the discharge were thoroughly characterized using voltage and current derivative monitoring techniques. A peak current of 15 kiloamperes was achieved within 110 nanoseconds in a short-circuit configuration at a 9 kV charging voltage. Plasma dynamics were captured through optical refractive diagnostics employing a pulsed Nd-YAG laser with a 170-picosecond pulse duration. Clear evidence of the z-pinch effect was observed during discharges in a deuterium atmosphere at 4 millibars and 6 kilovolts. The measured pinch length and radius were approximately 0.8 mm and less than 100 μm, respectively. Additionally, we explore the potential applications of this compact pulsed plasma source. These include its use as a plasma shock irradiation device for analyzing materials intended for the first wall of nuclear fusion reactors, its capability in material film deposition, and its utility as an educational tool in experimental plasma physics. We also show its potential as a pulsed plasma thruster for nanosatellites, showcasing the advantages of miniaturized plasma focus technology.

## 1. Introduction

The plasma focus has a special feature that is a self-scale kind of z-pinch [1]. This characteristic allows the reproduction of a physical scenario similar to that of small experimental devices, as opposed to the one obtained in huge plasma focus facilities. Fifty years ago, and during the first three decades, the plasma focus was studied as a possible device to produce dense transient plasmas for thermonuclear fusion research. The trend was to produce bigger devices over MJ stored energy and MA current through the plasma in order to increase the efficiency of fusion neutron production [2]. Unfortunately, neutron production suffers saturation in devices operating at MA [3,4]. However, the plasma focus is an interesting candidate for a pulsed plasma source and for a non-radioactive generator of X-rays and neutrons [5,6].

**Figure 1:**
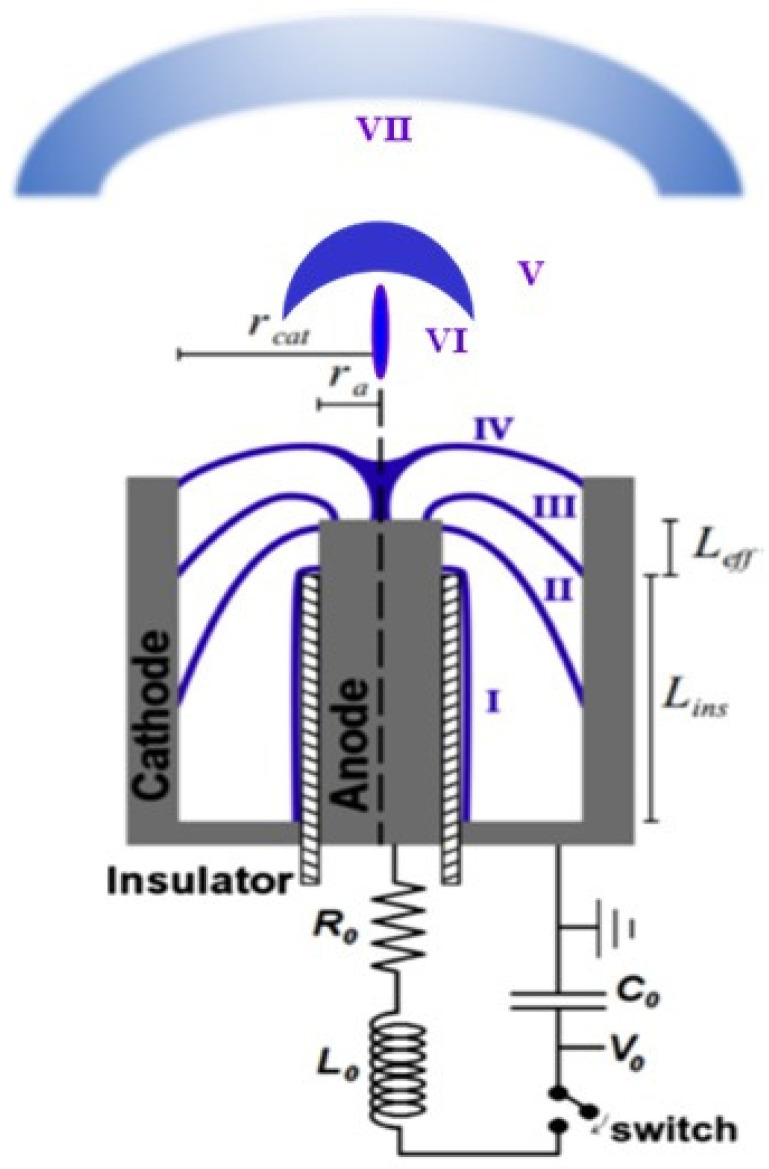
Scheme of a plasma focus and its plasma dynamics. Cathode radius: r_cat_, anode radius: r_a_, effective anode length L_eff_ = z_a_, and insulator length: L_ins_. Capacitance of the capacitor bank: C_0_, charging voltage: V_0_, total resistance: R_0_, and total inductance: L_0_. The anode and cathode are coaxial and are separated by an insulator. (I) The discharge starts over the insulator. (II) The Lorentz force produced by the current and the self-generated magnetic field pushes the plasma sheet to move axially, (III) then to move radially (sometimes plasma filaments appear), (IV) the sheet collapses to form a dense column of plasma (pinch), during these stages, electron beams, ion beams, X-rays, and neutron pulses (when operating with deuterium) are generated, (V) after the pinch is disrupted an axial shock is produced and thus, (VI) plasma jets are ejected, (VII) a cooler and less dense plasma reaches farther from the anode.

The plasma focus (PF) [1,2,5,7] is a type of pinch discharge in a gas at pressures of a few millibars. In Figure 1, a scheme with the description of a plasma focus is presented. A high-pulsed voltage is applied across two coaxial cylindrical electrodes, separated by an insulator. The discharge initiates over the insulator surface. Subsequently, the plasma sheath detaches, accelerated axially by the magnetic field self-generated by the current. Upon traversing the upper end of the central electrode (anode), the plasma becomes compressed in a compact region known as the focus or pinch. This pinch then disrupts, propelling the plasma axially as a shock wave [8], followed by the emanation of plasma jets from the anode region [9,10,11,12,13]. During certain stages, radial filaments are discernible [14,15].

The dynamics presented by a plasma focus device [1,2,5,7] are intricate. Interactions among plasma, electric fields, and magnetic fields lead to diverse phenomena, including magnetic pinches, plasma shocks, instabilities, pulsed particle and radiation emissions (X-rays, ion and electron beams, neutron bursts), and plasma jets. The initial electrical gas breakdown on the insulator [16,17] sets pivotal conditions for subsequent plasma sheath formation and movement. An irregular breakdown can detrimentally affect plasma dynamics and the resultant focus. The temporal correlation between the spark gap and insulator breakdown offers valuable data for studying non-conventional statistical distributions. Occasionally, during the breakdown phase, areas of anomalous resistance can emerge, potentially impeding the optimal formation and motion of the current sheath—a critical element for the plasma pinch effect. As plasma interacts with the insulator, charge accumulations may arise on its surface, potentially introducing unexpected electric fields that disrupt the intended plasma dynamics. Prolonged interactions can lead to insulator erosion, introducing plasma contaminants.

During the axial phase, the plasma sheath attains speeds of approximately ~10^5^ m/s at the end of the anode, with potential manifestations of toroidal singularities and filaments [14,15]. In the radial phase, the plasma sheet reaches velocities of roughly ~2.5 × 10^5^ m/s, forming a dense plasma column—the pinch—with particle densities around ~10^25^ m^−3^ and temperatures ranging from 0.5 to 1 keV. Filaments might become visible in this phase. Concurrently and post-pinch, emissions of X-rays, ions, electron beams, and, in deuterium discharges, neutron bursts are evident. Instabilities may also emerge during the pinch. Following the pinch, plasma ejection occurs axially, generating an axial plasma shock with speeds approximating ~5 × 10^5^ m/s [8]. This is succeeded by the formation of narrow plasma jets [9] with velocities around ~0.4 × 10^4^ m/s. Ultimately, a cooler and less dense plasma reaches farther from the anode.

The plasma focus thus presents a wealth of phenomena ripe for fundamental research, including the study of plasma instabilities, high-energy radiation production, fusion reactions, shock wave dynamics, magnetic reconnection, and anomalous resistivity [18]. Furthermore, it serves as a laboratory-scale model for astronomical phenomena [9,10,11,12,13] (using the plasma shock and plasma jets of phases V and VI in Figure 1) and offers insights into non-conventional statistical distributions and complex systems. In addition, given the extreme and transient nature of the plasma focus, there is always a need for advanced diagnostics [19,20]. This pushes the boundary in developing tools to measure plasma properties under challenging conditions.

Moreover, the plasma focus stands out as a versatile pulsed radiation source, finding utility in diverse scientific disciplines and myriad applications. In the realm of material science, it aids in plasma deposition on materials, nanomaterial fabrication (using the cooler and less dense plasma reaches farther from the anode, phase VII in Figure 1), and lithography (using X-rays and electron beams) [21,22,23,24,25,26,27]. It offers a platform to subject materials to extreme radiation conditions, acting as a plasma accelerator for analyzing materials under fusion-relevant pulses (using the axial plasma shock, phase V in Figure 1) [8,28,29,30,31,32] for the first wall of future thermonuclear fusion reactor. In the fields of biology and biomedicine, the plasma focus serves as an unparalleled radiation source for studying the effects of low total dose but high transfer rate pulsed radiation on living matter. Such studies encompass ultra-flash cancer therapy, radiobiology, and pulsed dosimetry, leveraging the unique capability of plasma focus to produce high-energy X-rays and neutrons in the nanosecond range [33,34,35,36,37,38]. In aerospace engineering, there is ongoing development of pulsed plasma thrusters for nanosatellites using plasma focus technology [39,40,41]. Furthermore, the device finds applications in flash radiography and substance detection and serves as a portable radiation source in field applications [42,43,44,45]. Various other potential uses are continually being researched and explored.

For decades, plasma focus devices were designed and constructed to operate with energies ranging from megajoules to kilojoules, with sizes from hundreds to a few cubic meters. Due to the scalability properties of plasma focus devices, plasma focus experiments have recently been extended to sub-kilojoule devices and scaling laws have been extended down to regions of less than one joule [1]. In the last two decades, several studies have been conducted on plasma foci operating at a few kilojoules to test possible applications of pulsed X-rays and neutrons in laboratory conditions. In the early 2000s, a new generation of plasma focus devices below kilojoules started with the idea to produce portable devices, non-radioactive X-rays, and neutron sources for field applications [46,47,48]. Presently, there are very small plasma focus devices operating worldwide in the energy sector under 1 kJ [46,47,49,50,51,52], under 100 J [48,53,54,55,56,57,58,59,60,61,62], and even below 1 J [63,64,65,66]. The efforts in the miniaturization of plasma focus devices are for both basic studies and technological applications. These studies have contributed to learning that it is possible to scale the plasma focus on a wide range of energies and sizes, keeping the same value of ion density, magnetic field, plasma sheath velocity, Alfvén speed, and temperature. However, the plasma stability depends on the size and energy of the device [1].

In this work, a miniaturized 2-Joule pulsed plasma source using plasma focus technology is presented. The electrical characterization of the discharge was performed using voltage and current derivative monitoring techniques. The plasma dynamics in this extremely miniaturized plasma focus device were captured through optical refractive diagnostics employing a pulsed Nd-YAG laser with a 170-picosecond pulse duration.

Finally, we explore potential applications of this compact pulsed plasma source. This encompasses its capability as a plasma shock irradiator for analyzing materials intended for the first wall of nuclear fusion reactors, for material film deposition, and as an educational tool in experimental plasma physics. Additionally, it can serve as a pulsed plasma thruster for nanosatellites, leveraging miniaturized plasma focus technology. The applications as plasma shock irradiators and pulsed plasma thrusters for nanosatellites are in progress, and therefore, details and more results will be part of future publications.

## 2. Materials and Methods

### 2.1. The Apparatus

Drawing from the knowledge gained in the scalability of plasma focus devices [1,5,42,43,48,53,55,63,64,65,66], we designed and built a compact, portable unit. This unit is engineered to operate at low kilovolts (up to 9 kV) and stores an energy of a few joules. The device PF-2J, including the capacitor, spark gap, discharge chamber, and power supply, is portable in hand luggage and has a total weight of less than 1.5 kg. The present version uses a home-made capacitor of a capacitance C = 120 nF. To obtain low inductance, the capacitor was connected in a compact layout. Thus, a short and coaxial spark gap was designed for that purpose, and the capacitor was connected directly to the spark gap and the electrodes. The spark gap operates in the open air. The measured total external inductance L is 40 nH. The total impedance of the generator is in the order of 0.58 Ω. The device operates at voltage V from 6 to 9 kV charging voltage (i.e., stored energy of ~2–4 J). The maximum current I ranges from 10 to 15 kA at short circuit. Table 1 summarizes the electrical parameters of the PF-2J device.

Electrode dimensions were determined from the design relations and scaling rules presented in reference [1]. For plasma foci operating in deuterium at a few mbar and optimized for neutron emission, it has been observed in a wide range of energies and sizes that for a capacitor bank charged at an energy E, the following relation for the energy density parameter (28 E/*a*^3^) ~5 × 10^10^ J/m^−3^ is satisfied (with *a* as the anode radius) [1,53]. Thus, for a PF operating in the range 2–4 J, *a* is in the order of 1 to 1.3 mm. The effective anode length, z_a_, must be such that the pinch occurs close to the peak current. The time from the beginning of the current up to the maximum compression, t_p_, includes t_p_ = t _I_ + t_a_ + t_r_, where t _I_ is the time that the current sheath is kept on the insulator, t_a_ is the time of the axial phase of the current sheath, and t_r_ is the time of radial phase of the current sheet. According to experimental observations and from the analysis of the electrical signals in small and fast PF discharges, t _I_ is of the order of T/12, with T in the first period of the discharge [67,68,69].

As the time to the maximum compression must be close to the first quarter of the period of the discharge, the effective length can be estimated from T/4 = t_p_, i.e., T/4 = T/12 + *a*/<v_r_> + z_a_ <v_a_>, with <v_a_> the mean axial velocity and <v_r_> the mean radial velocity.

Using the measured T = 440 ns, choosing *a* = 1.1 mm and considering that the mean axial velocity is <v_a_> ~5 × 10^4^ m/s and the mean radial velocity is <v_r_> ~1.25 × 10^5^ m/s [1,68], an estimated value for z_a_ ~3.3 mm is found. These values are a guide to constructing a plasma focus with a capacitor bank of 120 nF with a total external inductance of 40 nH operating at ~2 J. However, the dimensions must be optimized experimentally.

According to our experience, the length of the insulator should be in the order of ~1–1.5 mm per kV of charging voltage.

**Figure 2:**
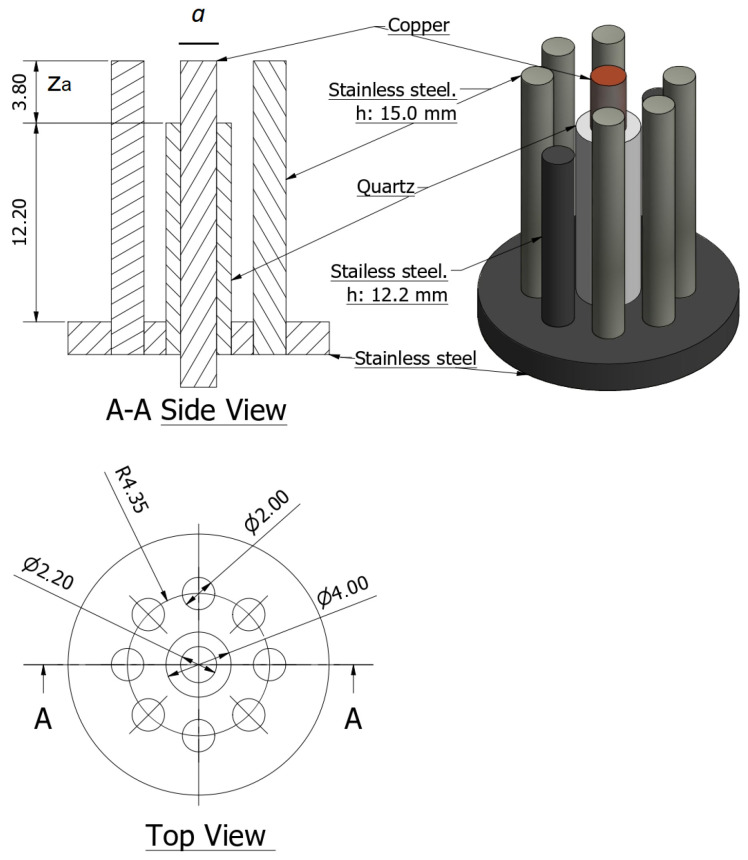
Electrode configuration diagram (in mm). (**Left**): top: side view, bottom: top view. (**Right**): 3D diagram. The anode is the central electrode, an insulator covers part of the anode, and the cathode is the eight rods around the anode. Two opposing rods are shorter to provide an adequate field of view for optical diagnosis.

Finally, the actual miniature plasma focus constructed has the following dimensions and features: (a) the electrode structure consists of a 15 mm long, *a* = 1.1 mm radius copper tube anode and an outer cathode of eight 2 mm diameter stainless steel rods uniformly spaced on an 8.7 mm diameter (six of the cathode rods have a length of 15 mm and two opposite rods have 12.2 mm length to provide a proper field of view for optical diagnostics), (b) the anode and cathode were separated by a quartz tube of 12.2 mm length, resulting in an effective anode length of z_a_ = 3.8 mm. It is noticed that plasma focus devices are expected to produce a hot, dense plasma during the pinch; therefore, the cathode configuration rods are used instead of a cylinder to reduce the impurities from the cathode that could cool the plasma generated from the filling gas. Figure 2 shows a diagram of the electrode configuration.

**Figure 3:**
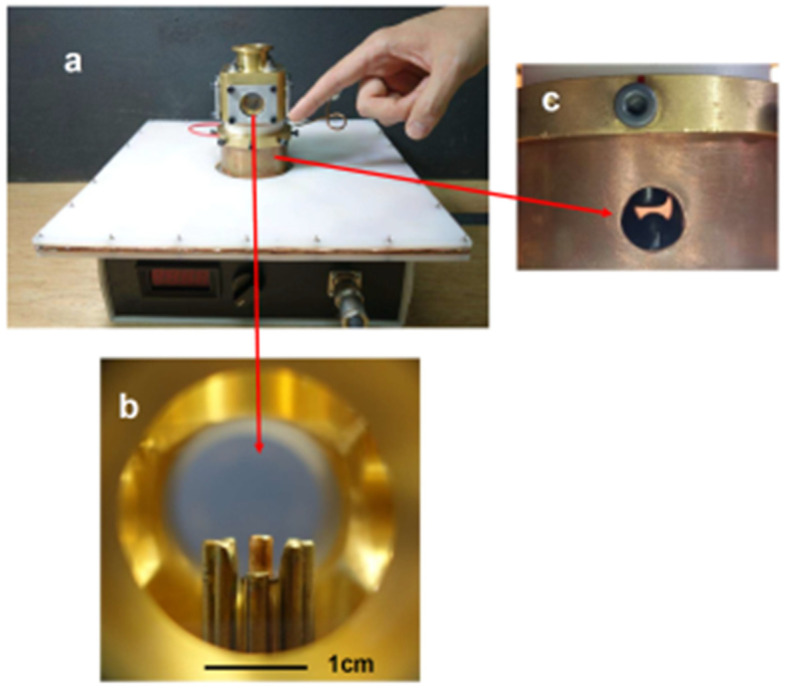
(**a**) The device, including the power supply, with the dimension of the square white capacitor of 31 cm × 31 cm × 31 cm with a thick of 16 mm. (**b**) Electrode configuration with the 2.2 mm diameter anode seen at the center. (**c**) A view of the spark gap, the gap between the electrodes is 2.8 mm for a discharge at 8 kV.

In order to obtain images from the plasma, a discharge chamber with four windows was designed and constructed. Figure 3 shows (a) the device, including the power supply, (b) the electrode configuration inside the discharge chamber as seen from a window, and (c) a view of the spark gap.

### 2.2. Experimental Characterization

#### 2.2.1. Electrical Diagnostics

Monitoring the voltage, V(t), current, I(t), and current derivative, dI(t)/dt, signals in a plasma focus offers a direct insight into the operation of the device and the behavior of the plasma [70]. This makes it an essential diagnostic tool for both operational and research activities. These measurements provide valuable information about the electrical characteristics and dynamics of the plasma. Specifically, the current derivative and the current waveform are closely related to various stages of plasma dynamics, including the axial phase, radial phase, and pinch phase. The pinch phase is marked by a dip in the current derivative, accompanied by a sharp rise in the current. Recognizing the duration and magnitude of these changes is vital for optimizing neutron and X-ray yields. Concurrently, with the dip in the current derivative, a voltage peak emerges. This distinct signature in the signals corresponds to a significant change in the inductance of the discharge, resulting from the high radial compression velocity during the pinch effect. For consistent operation and to achieve desired outcomes (like maximum neutron yield), it is crucial to ensure the reproducibility of voltage and current signals for each shot. Monitoring these parameters allows for better control and optimization of the device. In addition, monitoring the voltage and current can also be essential for safety reasons. If the voltage or current signals deviate from the signatures, it can mean operational anomalies or malfunctions. These anomalies include, but are not limited to, disruptions in the plasma generation process, damage to the electrical components of the devices, decreased efficiency in plasma focus operation, and safety concerns for operators due to erratic device performance. Identifying deviations in voltage or current signatures allows for early detection and mitigation of these problems, ensuring the device operates safely and effectively.

The voltage monitor, a voltage divider with a factor of 1000 and a bandwidth of 150 MHz, was connected to the central anode rod, positioned after the spark gap and near the plasma load.

A Rogowski coil was employed for current and derivative measurements. This is a well-known and versatile tool often used for current measurements, especially when there is a need for wide bandwidth or recording high transient currents [71,72]. In our setup, the Rogowski coil, once calibrated, monitored the current derivative signal dI(t)/dt of the circuit around the base of the anode. The current I(t) is obtained by integrating numerically dI(t)/dt.

Both the voltage divider and Rogowski coil signals were captured using a Tektronix, model TDS 3054C digital oscilloscope, Beaverton, OR, USA (4 channels, 500 MHz, 5 GS/s).

#### 2.2.2. Optical Refractive Diagnostics

Optical refractive diagnostics, leveraging the principles of refractometry, offer a non-invasive and highly sensitive approach to probe and record plasma dynamics. As plasma undergoes changes in temperature, density, or composition, its refractive index varies, which can influence the propagation of light passing through it. For dense plasmas such as z-pinches, plasma foci, laser-produced plasmas, and others, the refractive index correlates directly with the density [73].

By directing a laser or coherent light source through the plasma and analyzing the resulting light distortions or deflections in the image produced in a screen, film, or CCD camera, valuable information about the internal structures of the plasma, wave phenomena, and turbulence is possibly inferred. Typically, the light is analyzed using shadowgraphy, schlieren, or interferometry techniques [73]. In shadowgrams, the intensity difference in the image is proportional to the second spatial derivative of the refractive index. In schlieren imagery, it is proportional to the spatial derivative of the refractive index. Meanwhile, for interferograms, information about the refractive index can be extracted from phase change associated with the shifts of the fringes.

**Figure 4:**
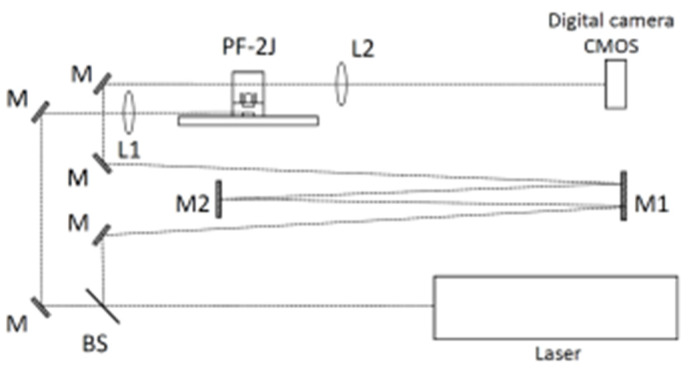
Optical setup. M, M1, M2: mirrors, BS: beam-splitter. The BS splits the laser beam; one is used to trigger the spark gap, and the second to produce an image of the plasma. Adjusting the distance between M1 and M2 and the number of reflections, the time of flight for the image of the plasma dynamics is selected. L1 and L2: lens. PF-2J: plasma focus. To trigger the spark gap, one of the beams is focused (with the lens L1) on the bottom electrode of the spark gap. The lens L2 produces an image of the plasma over the CMOS.

Figure 4 shows the optical setup used to study the plasma dynamics by means of an optical refractive diagnostic. A regular bi-convex lens (L2 in Figure 4) with a focal length of 20 cm and an optical number F = 1/10 was used to produce the plasma image on a CMOS. A shadowgram-type optical system based on a slightly out-of-focus image-plane setup was implemented. In this setup, the imaging lens (L2) is slightly displaced from the correct focus position (a few millimeters), producing an image of the plasma refractivity effect on the scene beam in a plane posterior to the plasma position.

In the PF-2J plasma focus, the time history of the plasma dynamics is in the order of 150 ns and larger. To obtain an image of the plasmas by means of an optical refractive diagnostic, a single pulse laser of 170 ps is synchronized into the time history of the discharge.

A pulsed Nd-YAG laser (Ekspla, model SL 334/SH, Vilnius, Lithuania) of 170 ps pulse duration at 532 nm was used. The image acquisition was achieved using a digital camera (Canon, model EOS Rebel T2i, Tokio, Japan) with a CMOS size of 14.8 mm × 22.2 mm (5.2 μm pixel size). A magnification m = 3 was used; thus, one pixel corresponds to 1.7 µm.

To synchronize the laser pulse into the time history of the plasma discharge, the laser beam was split into two beams: one was used to trigger the discharge by focusing the beam (with the lens L1) on one of the spark gap electrodes, and the other one was used to produce the image of the plasma discharge (with the lens L2) after some time of flight (for example 3 m correspond to 10 ns of time of flight). Adjusting the distance between M1 and M2 and the number of reflections, the time in which the pulse laser passes through the plasma is used to obtain the image. One image per shot for a particular time of the discharge is recorded.

## 3. Results

**Figure 5:**
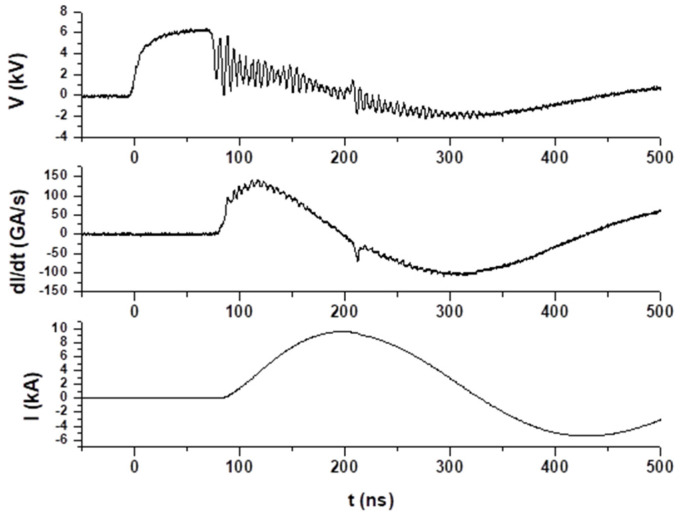
Electrical signals in a discharge at 4 mbar in deuterium and 6.3 kV charging voltage, a dip in the current derivative, and a peak in the voltage appear 132 ns after the initiation of the discharge; both are evidence of the occurrence of a pinch. A maximum current of 9.6 kA was achieved at 118 ns after the initiation of the discharge.

Discharges were performed at 6–8 kV charging voltage in deuterium with pressures ranging from 1 to 20 mbar. After some hundred shots, a pinch was observed in the electrical signals at pressures in the range of 3 to 6 mbar. Figure 5 shows electrical signals in a discharge at 4 mbar in deuterium and 6.3 kV charging voltage. A maximum current of 9.6 kA was achieved at 118 ns after the initiation of the discharge. The typical dip in the current derivative signal and the peak in the voltage signal associated with the formation of a pinched plasma column on the axis are clearly observed. The minimum value of the current derivative was achieved at 132 ns after the initiation of the discharge.

**Figure 6:**
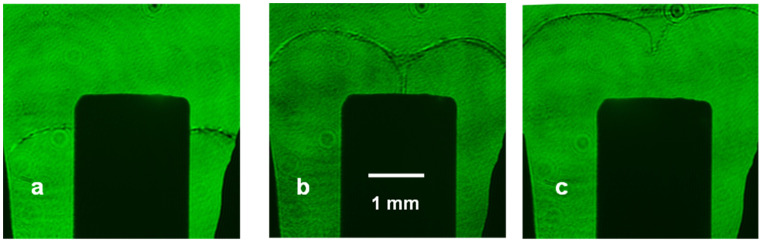
Images of the plasma dynamics: (**a**) the axially moving plasma sheath (~100 ns after the current started), (**b**) the pinch moment (~140 ns after the current started), and (**c**) after the pinch disruption (~150 ns after the current started).

Figure 6 shows the shadowgrams obtained. Three phases of the plasma dynamics are clearly distinguished: (a) the axially moving plasma sheath, (b) the pinch moment, and (c) after the pinch disruptions. The observed pinch length and radius are ~0.8 mm and ~100 μm, respectively. In addition, a thickness for the current sheath of <100 µm is observed from the images, and the formation of instabilities is not evident from the image of the pinch phase.

## 4. Discussion

A miniature plasma focus device operating at 2 joules was constructed using the scaling rules [1,74] and experimental observations collected for several plasma foci operating in a wide range of energy [1]. In the constructed device, the electrical signals associated with a pinch were obtained, and the typical plasma focus dynamic was observed using optical refractive diagnostics. The typical dip in the current derivative and the peak in the voltage signals associated with the formation of a pinched plasma column on the axis were observed. From the shadowgrams, three phases of the plasma dynamics were distinguished: (a) the axial phase, (b) the pinch, and (c) pinch disruptions. The size of the pinch confirms the expected value from scaling rules (i.e., pinch length ~(0.8–1) × *a*, pinch radius ~(0.1–0.2) × *a*, with *a* as the anode radius) [1,74]. Thus, the design methodology used here was experimentally confirmed as a useful tool.

The reported device, PF-2J, includes the capacitor, spark gap, discharge chamber, and power supply, and is portable in hand luggage with a total weight of less than 1.5 kg. In addition, due to the low energy operation per shot (~2 J), it is possible to operate the device at a repetition rate without a cooling system. At 1, 10, and 100 Hz, the average power is in the order of 1 to 2, 10 to 20, and 100 to 200 W. These characteristics allow the use of the device in different research and exploration of applications in which a compact size and weight could be an advantage or the repetition rate is required.

A characterization of its statistical reproducibility and reliability analysis is indispensable for any application. In previous work, a statistical study and reliability analysis program for the tabletop plasma focus device PF-2J was conducted based on the analysis of the dI/dt(t) and V(t) signals in relation to the accumulated number of shots [75]. It was found that the life cycle for the pinch plasma voltage and the amplitude of the dip in the current derivative signal exceeds 10^4^ shots, without significant changes in the pinch voltage and with only a 10% decrease in the amplitude of the dip in the current derivative [32,75,76].

Thus, related to the utility of this kind of compact portable pulsed plasma device, some of their training in experimental plasma physics, plasma deposition on materials and nanomaterial fabrication, pulsed plasma sources to study materials for the first wall of nuclear fusion devices, and pulsed plasma thruster for nanosatellites orientation are noticed.

### 4.1. Training in Experimental Plasma Physics

Previous versions of this device [42,43,77] were transported in hand luggage from Chile to Italy, to the International Center for Theoretical Physics, ICTP, Trieste, and practical training lessons were conducted using the PF-2J at ICTP during the School and Training on Dense Magnetized Plasmas in 2010 and 2012. In 2018, a version of the present device was used at the Joint ICTP-IAEA College on Plasma Physics [78].

### 4.2. Plasma Deposition of Materials and Nanomaterial Fabrication

Plasma foci have been widely used to produce film deposition of materials and nanomaterial fabrication from the interaction of the low-dense and low-temperature plasma in region VII in Figure 1 [21,22,26,27]. The difference with a small device is the size of the area in which it is possible to deposit material. Even though the area is the smallest, it is enough to produce new materials and study its properties. In fact, a device of only 50 joules was successfully used to study Ti film deposition [22]. Moreover, an extremely miniaturized plasma focus operating at only 0.1 J [63,64,65,66] has been preliminary used to produce films.

### 4.3. Pulsed Plasma Sources to Study Materials at Extreme Conditions: First Wall of Nuclear Fusion Devices

In selecting materials for plasma-facing components in nuclear fusion reactors, such as ITER for magnetic fusion energy (MFE) and experiments like those at the National Ignition Facility for inertial fusion energy (IFE), it is crucial to simulate the extreme conditions these materials will face. This includes handling intense heat and particle fluxes. In MFE, energy loads might reach 100–300 J/cm^2^ over 0.1–0.5 ms with up to 10^3^ pulses per shot, while IFE conditions can see 3–6 J/cm^2^ over 0.2–1 μs with a frequency of about 5 Hz, showcasing the varied and extreme testing environments for these materials. Therefore, it is highly desirable to have a universal or equivalent measure of radiation damage that characterizes the surface erosion in all these different environments. The equivalence is established using a practical parameter called damage factor, F ~ q × τ^1/2^ (with τ the interaction time of the radiation with the material and q the heat flux deposited on the material) [29,30]. Research indicates that materials exposed to high-power radiation flux for short durations exhibit similar thermomechanical effects to those irradiated with lower power but for extended periods, provided the damage factor remains constant [29]. This concept is exemplified by tungsten subjected to varying radiation conditions across different facilities, showing melting at consistent damage factor levels. For instance, melting in tungsten was noted under diverse irradiation scenarios: at the RHEPP ion accelerator with a 4.5 J/cm^2^ exposure for 200 ns, via the QSPA Kh-50 plasma gun with 150 J/cm^2^ for 0.5 ms [79], and at the JUDITH electron accelerator with 550 J/cm^2^ for 3 ms. In each instance, the damage factor, F, ranged from 0.7 × 10^4^ to 1 × 10^4^ (W/cm^2^)s^1/2^. These observations align with the expected damage factor F for ITER and IFE experiment reactor walls, approximately 10^4^ (W/cm^2^)s^1/2^. A theoretical model posits that this damage factor directly reflects the peak concentration of radiation-induced defects in materials, emphasizing the energy required for defect formation over the type of irradiation [30].

It has been shown that the axial plasma shock after the pinch observed in tabletop plasma foci operating at a hundred joules can produce damage on materials equivalent to that expected in the first wall of Inertial Fusion Energy (IFE) devices and in Magnetic Fusion Energy devices [8,28,80]. PF devices can produce F in the order of ~10^4^ (W/cm^2^)s^1/2^ and greater [8,28,30,31,32]. As previously mentioned, this is the order of magnitude of the damage factor expected on the first walls of Inertial Fusion Energy (IFE) devices and Magnetic Fusion Energy (MFE) devices.

Using the scaling rules for the damage factor, F, with plasma foci energy, E, a tunable damage factor pulsed plasma shock irradiator device based on the PF-2J was designed and constructed (F scale as E^1/6^ [32]). This relation, F scales as E^1/6^, is obtained from scaling rules for size, velocity, and density for the pinch in different PF devices operating at stored energies from less than 1 J to MJ [1]. This means that if we consider the damage factor for a PF with 1000 J stored energy as F, PF devices operating with stored energies of 1 kJ, 100 J, 10 J, and 1 J will have damage factors of 1/3 F, 1/5 F, 1/7 F, and 1/10 F, respectively. Roughly speaking, the damage factor for the PF-1000 (1 MJ) in Poland is only 3.65 times greater than the damage factor for the PF-400J (400 J) and 8.9 times greater than the damage factor for the PF-2J (2 J), both in Chile. The PF-2J produces a damage factor that is roughly 1/4 of the damage factor produced by the PF-400J. A manuscript describing the scaling rule for F depending on the stored energy is in preparation. On the other hand, the damage factor F has a strong dependence on the distance to the target; thus, a micrometric positioner for the material samples was designed and included [76]. Thus, the PF-2J device produces a damage factor, F, ranging from 10^2^ to 10^4^ (W/cm^2^) s^1/2^ [32].

**Figure 7:**
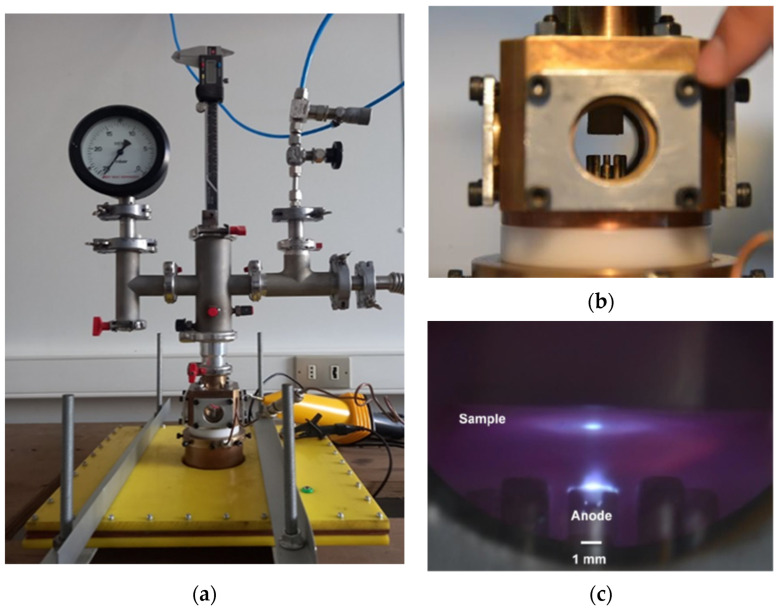
(**a**) The whole device, including the X, Y, and Z positions for materials samples. In the axis, Z is a micropositioner. (**b**) Discharge chamber details, cathode bars, and the anode at the center. Over the anode is the sample holder, which is axially adjusted with the micropositioner. (**c**) Time-integrated photograph of a single discharge in the PF-2J used as the pulsed irradiator. Plasma is seen on the top of the anode (anode diameter 2.2 mm). Also, a bright spot is seen in the sample located over the anode due to the axial plasma shock interacting with the sample.

Figure 7a shows the PF-2J, including the micrometric positioner for the material samples, Figure 7b shows details of the discharge chamber and electrodes, and Figure 7c shows a time-integrated photograph of a single discharge in the PF-2J used as the pulsed irradiator.

It allows for a repetition rate of ~0.1 Hz, enabling material irradiation with 10, 100, and 1000 shots in 100 s, 20 min, and 3.2 h, respectively. In contrast, larger facilities such as the RHEPP ion accelerator, QSPA Kh-50 plasma gun, and JUDITH electron accelerator can study only a limited number of samples and obtain a few shots per day.

The repetitive tabletop pulsed plasma shock irradiator presented here was used to test SS samples (AISI 304) at different positions from the anode top: 2.8, 3.6, and 5.4 mm, producing a damage factor F of approximately 10^4^, 10^3^, and 10^2^ (W/cm^2^)s^1/2^ per shot, respectively [32]. At 2.8 mm, i.e., F ~ 10^4^ (W/cm^2^)s^1/2^ per shot, 1, 10, 100, and 1000 shots were accumulated in SS samples [32].

**Figure 8:**
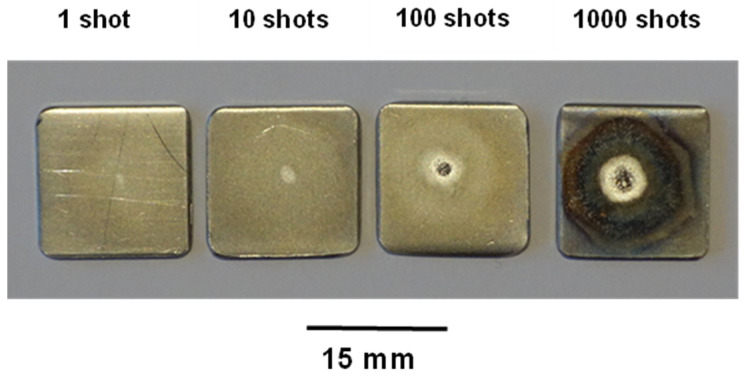
SS samples (AISI 304) irradiated with the PF-2J using 1, 10, 100, and 1000 shots at ~ 0.1 Hz. Samples were located 2.8 mm from the anode top (F~10^4^ (W/cm^2^)s^1/2^. From Figure 11 of Ref. [32].

Figure 8 shows SS samples (AISI 304) irradiated with the PF-2J at ~0.1 Hz. Samples were located 2.8 mm from the anode top (F~10^4^ (W/cm^2^)s^1/2^ per shot) and were irradiated with 1, 10, 100, and 1000 shots [32].

### 4.4. Pulsed Plasma Thruster for Nanosatellite Orientation

Axially ejected plasma shocks and plasma jets have been observed in plasma focus devices, leaving the electrodes at high velocity. For instance, in a plasma focus operating at 400 J, a plasma of ~10^−10^ kg is ejected from the pinch with a velocity >10^5^ m/s. These plasma conditions appear promising to be used as the base of a pulsed plasma thruster (PPT), particularly to develop a miniaturized propulsion device for orientation, capable of being integrated into a small-standardized satellite, such as the CubeSat.

To explore the possibility of using our experience in the scaling and miniaturization of plasma focus devices, a PF device designed and constructed in our laboratory, the Nanofocus, that operates at an energy level of tenths of joules was modified to be used with different configurations of plasma guns (Nanofocus, 5 nF, 5 nH, 1–10 kV charging voltage, 2.5–250 mJ stored energy, 1–10 kA maximum current [63,66]). It is important to take into consideration that a plasma focus works at millibar pressures, and a PPT works in a space environment, i.e., vacuum; thus, in the PPT, the plasma will be generated from the ablation of the insulator material, PTFE for example. For that reason, a pressure lower than 10^−4^ mbar was used to operate the modified Nanofocus.

**Figure 9:**
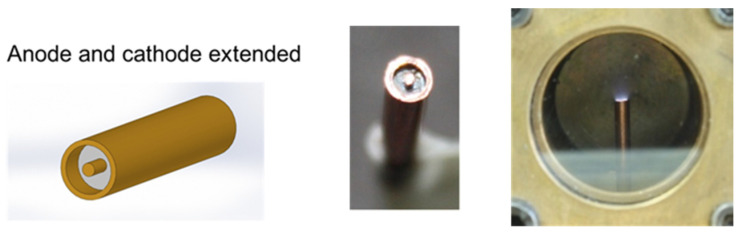
Coaxial plasma gun with cathode and anode extended. External cathode radius: 1.1 mm, Internal cathode radius: 0.85. Left and center: photograph of plasma gun. Right: time-integrated photograph of the plasma discharge. The voltage breakdown was 1.75 ± 0.2 kV, and the maximum current was 0.6 ± 0.1 kA.

**Figure 10:**
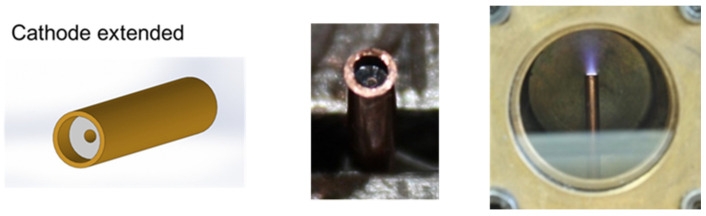
Coaxial plasma gun only with cathode extended. External cathode radius: 1.1 mm, Internal cathode radius: 0.85. Left and center: photograph of plasma gun. Right: time-integrated photograph of the plasma discharge. The voltage breakdown was 2.2 ± 0.1 kV, and the maximum current was 0.8 ± 0.1 kA.

Thus, experiments using a modified ultraminiaturized plasma focus device as the core element of a PPT were developed. Different geometries of electrodes (plasma guns) were studied and evaluated, producing a hundred discharges for each configuration. These conceptual experiments were operated at 0.1 J, producing electrical discharges at 10^−4^ mbar on plasma guns with submillimeter internal and external radii. Figure 9 and Figure 10 show results obtained for two different plasma guns. The dimensions of the coaxial electrodes used in the experiments were as follows: External cathode radius: 1.1 mm, Internal cathode radius: 0.85 mm, and anode radius: 0.35 mm. Teflon (PTFE) (RS Inc., Fort Worth, TX, USA) was used as a solid propellant.

A difference in the voltage breakdown, maximum current, and plasma images was observed between both plasma gun configurations. In the case of the coaxial plasma gun with the cathode extended (Figure 10), the plasma ablated from the PTFE material was ejected axially at a smaller solid angle than the case of the electrode configuration of Figure 9. Thus, the electrode configuration with the cathode extended is more proper as a pulsed plasma thruster than the electrode configuration with the cathode and anode extended. These results were presented at two international conferences [40,41].

These results using a pulsed generator of less than one joule encourage us to explore the possibilities of using our experience in the scaling and miniaturization of plasma focus technology to develop a miniaturized pulsed plasma thruster for nanosatellites.

Scaling estimations are useful in supporting this work. According to theoretical and scaling estimations, it is expected that for a pulsed plasma thruster operating with a stored energy of 1 J, a bit impulse in the range of fractions of μNs to some μNs per pulse would be obtained. On the one hand, to obtain an estimation for a miniature plasma thruster operating with an energy of the order of 1 J, based on plasma focus technology and its scaling laws, we can assume an ejected mass m_e_ ~ 4 × 10^−13^ kg with a velocity v_e_ ~ 5 × 10^5^ m/s. Thus, an impulse bit I_bit_ = Δp = m_e_ v_e_ ~ 2 × 10^−7^ Ns is estimated. Considering that the mean propulsion force during a second is <F> = I_bit_ f, with f the operation frequency, the mean thrust could be 0.2, 2, and 20 µN for an operation frequency of 1, 10, and 100 Hz, respectively.

On the other hand, from electromagnetic estimation for a coaxial plasma gun (axial phase of a plasma focus), imposing the condition that the plasma reaches the end of the electrodes coincident with maximum current, i.e., at a time of quarter of the period of the discharge, Δp = F_mag_ (τ/4) = (µ_0_/4) I^2^ ln(b/a) (LC)^1/2^, with I the peak current, C de capacitance of the capacitor, L the total inductance, a the anode radius, and b the cathode radius. Using I = V (C/L)^1/2^, Δp = (µ_0_/4) V^2^ ln(b/a) C^3/2^ L^−1/2^. For a device with a capacitor of 225 nF charging at 3 kV, an energy storage of 1 J is achieved. Assuming a = 0.5 mm and b = 1.25 mm, and 5 nH of inductance (that is possibly achieved in compact devices like Nanofocus designed and built at CCHEN [63,64,65,66]), a value for Δp ~ 3.8 × 10^−6^ Ns is obtained. Thus, with 1, 10, and 100 Hz, the mean thrust could be 3.8, 38, and 380 µN, respectively. Both estimations are consistent with the literature for orientation systems for CubeSats [81,82].

Both estimations are consistent with the literature for orientation systems for CubeSats [81,82]; thus, using our experience in the miniaturization of plasma focus devices, a PPT to be integrated into a 1 unit CubeSat (10 cm × 10 cm × 10 cm and the typical weight of ~1 kg) is being presently developed. A compact capacitor of a capacitance of 1μF of low inductance to be charged at 1 to 2 kV, i.e., 0.5 to 2 J, has been constructed and is being characterized [40,41]. Several geometries of plasma guns of 1 to 2 mm of external diameter are being studied.

Also, a commercial small capacitor of 1.5 µF, 2 kV (3 J stored energy), 50 mm × 45 mm × 30 mm in size, and 115 g in weight, is being tested. This capacitor is charged using a voltage multiplier of 6 W, V_in_ = 12 V, V_out_ = 2 kV, with dimensions of 57 mm × 28 mm × 12 mm and a weight of 37 g. The plasma gun weighs approximately 2 g. Thus, the total weight and volume of the PPT system are around 150 g and 100 cm^3^. Although the number of shots required to achieve a specific orientation through small movements may be around 100 shots, this should suffice. At an operation rate of 1 Hz for each orientation, the PPT consumes approximately 300 J in 100 s, which corresponds to an average power of 3 W. A typical power supply for a nanosatellite of three CubeSat units is based on a battery of 24 V, 3 A, and 72 W. Generally, the lifetime of a nanosatellite of three CubeSat units is around 24 months. Therefore, the operation of a PPT for specific orientation purposes, as we are exploring, is feasible for CubeSat nanosatellites. The lifetime and material of the electrodes and the PTFE propellant are part of the current study as well. An idea of the reliability can be estimated considering that the plasma focus PF-2J device can operate for ~10^4^ shots [32,75,76].

At present, the impulse is being characterized in the laboratory under simulated space conditions. A thrust stand based on a single point load cell has been developed to measure impulses ranging from less than 1 μNs to tens of μNs.

In addition, compact design generators with low inductance and high current rise rates favor high input density power, which would allow more energy to be available to create the precursor plasma from the solid-propellant material [83,84].

## 5. Conclusions

In this study, we successfully constructed a miniature 2 Joule plasma focus device, guided by established scaling rules and empirical data from multiple plasma foci spanning a broad energy range. The device demonstrated expected electrical signatures linked to a pinch, and typical plasma focus dynamics were discerned through optical refractive diagnostics. This marks the first report of optical refractive diagnostics for a PF device of only 2 Joules, validating our design methodology as effective.

Such miniaturized pulsed plasma reactors hold significant promise in various applications, including experimental plasma physics education, plasma-enhanced material deposition, nanomaterial production, material studies for nuclear fusion reactor walls, and pulsed plasma thrusters for nanosatellite navigation. Specifically, a tunable damage factor pulsed plasma shock irradiator device based on the PF-2J was designed and constructed to study materials under extreme conditions. Additionally, a pulsed plasma thruster for nanosatellite orientation is being developed to integrate into a 1U CubeSat (10 cm × 10 cm × 10 cm) using the miniaturized plasma focus technology.

These applications are still in the research and development phase, with more detailed investigations planned for future publications. This study lays the groundwork for future developments, demonstrating the versatility and potential of compact, portable pulsed plasma sources.

## Figures and Tables

**Table 1:** PF-2J.

C (nF)	L (nH)	V (kV)	I (kA)	E (J)
120	40	6–9	10.4–15.6	2.16–4.86

## Data Availability

The original contributions presented in the study are included in the article, further inquiries can be directed to the corresponding author.
